# Tackling cuproptosis: from metabolic rewiring to therapeutic exploitation in cancer

**DOI:** 10.1038/s41423-026-01387-x

**Published:** 2026-01-27

**Authors:** Qian Hao, Yu Gan, Xiang Zhou

**Affiliations:** 1 Cancer Institute, Fudan University Shanghai Cancer Center, Shanghai, China; 2 Department of Oncology, Shanghai Medical College, Fudan University, Shanghai, China; 3 Key Laboratory of Breast Cancer in Shanghai, Department of Breast Surgery, Fudan University Shanghai Cancer Center, Shanghai, China

**Keywords:** Cuproptosis, Metabolism, Tumor microenvironment, Nanomedicine, Cancer therapy, Cancer metabolism, Targeted therapies

## Abstract

Cuproptosis, a recently identified copper-dependent form of regulated cell death, is driven by mitochondrial dysfunction caused by copper overload. Cuproptosis results from proteotoxic stress, which is triggered by copper-induced aggregation of lipoylated tricarboxylic acid (TCA) cycle enzymes and destabilization of iron-sulfur cluster proteins. This review elucidates the mechanisms of cuproptosis, emphasizing its regulation by copper homeostasis, metabolic reprogramming, and key signaling pathways such as p53, HIF-1α, Wnt/β-catenin, and AKT. Notably, copper modulates antitumor immunity through its effects on the tumor microenvironment, suggesting a critical role in cancer immunotherapy. Therapeutic strategies using copper ionophores and nanomedicine platforms demonstrate potential to induce cuproptosis in a variety of cancers. Preclinical studies highlight cuproptosis as a promising strategy against malignancies with copper dysregulation or mitochondrial metabolism adaptation, while clinical translation requires biomarker-driven patient stratification and optimized delivery systems. This synthesis provides a framework for harnessing cuproptosis in precision oncology, bridging mechanistic insights to therapeutic innovation.

## Introduction

Regulated cell death (RCD) is a crucial mechanism for living organisms to maintain homeostasis, coordinate development, and respond to pathological challenges. From the precise shaping during embryonic development to the dynamic balance of adult tissues, and to the abnormal clearance or malignant escape in disease states, different types of RCD execute their biological functions through unique molecular pathways [1–3]. Classic forms such as apoptosis, autophagic cell death, pyroptosis, necroptosis, and ferroptosis have been extensively characterized.

Apoptosis, a fundamental form of RCD, was first described by John Kerr, Andrew Wyllie, and Alastair Currie in 1972 [4]. It is characterized by specific morphological features, such as cell shrinkage, chromatin condensation, and the formation of apoptotic bodies. Apoptosis is driven by caspase-dependent cascades, playing critical roles in embryonic interdigital tissue remodeling, redundant cell elimination, and tumor suppression [5–8]. Autophagic cell death, involving lysosome-mediated self-degradation, balances cellular survival under nutrient stress, thus playing a role in the interplay between pro-survival and pro-death outcomes [9–12]. Pyroptosis is characterized in 2001 by Brad Cookson and Molly Brennan [13]. It is mediated by inflammasome-activated Gasdermin proteins that form membrane pores to cause cell lysis and passive release of pro-inflammatory cytokines (e.g., IL-1β), which amplifies immune responses against pathogens or tumor cells [14–19]. Necroptosis was first observed in 1996 by Caroline Ray and David Pickup [20], and the term was coined in 2005 by Junying Yuan and colleagues [21, 22]. It is activated mainly through the RIPK1/RIPK3/MLKL signaling axis, which features plasma membrane rupture and release of pro-inflammatory cytokines. It serves as both a defense mechanism against viral infections and a potential driver of inflammatory disorders [23–26]. Ferroptosis was first described in 2012 by Brent Stockwell [27], which is driven by iron-dependent lipid peroxidation and emerges as a focal point in tumor suppression and neurodegenerative pathologies [28–32]. In addition, other forms such as parthanatos [33], paraptosis [34], NETosis [35], entosis [36], and disulfidptosis [37] demonstrate context-specific functionalities in certain stress conditions or pathological settings.

Recently, the discovery of a novel RCD modality, cuproptosis, has revolutionized this field [38, 39]. Studies reveal that excessive copper ions directly bind to key enzymes in the mitochondrial tricarboxylic acid (TCA) cycle, such as lipoylated dihydrolipoamide S-acetyltransferase (DLAT). This binding induces aggregation of lipoylated proteins and mitochondrial dysfunction, ultimately leading to cell death. This mechanism operates independently of classical RCD pathways and is closely associated with copper metabolism dysregulation in human diseases.

This review will comprehensively elucidate the roles of cuproptosis in tumorigenesis and diverse pathological conditions by focusing on four pivotal dimensions: copper ion homeostasis, metabolic reprogramming, cuproptosis-associated signaling pathways, and tumor microenvironment. We will dissect how dysregulated copper metabolism synergizes with altered glycolytic flux to fuel cuproptosis susceptibility, particularly in malignancies characterized by mitochondrial dependency. Furthermore, we will explore the therapeutic potential of triggering cuproptosis, including strategies to modulate copper bioavailability (e.g., copper ionophores and nanomedicine) and combinatorial approaches leveraging metabolic vulnerabilities. By integrating mechanistic insights with translational perspectives, this essay aims to chart a roadmap for harnessing cuproptosis as a potential strategy against copper-associated diseases, including cancer.

## Copper metabolism

**Fig. 1:**
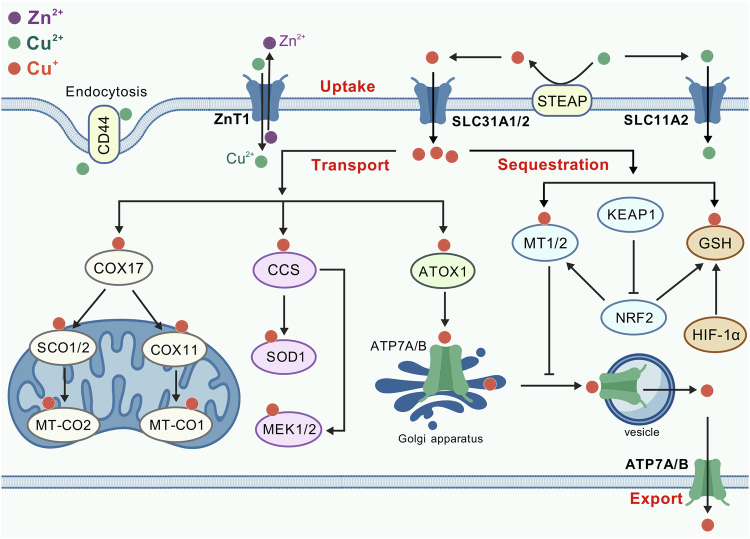
Copper metabolism. Copper homeostasis is tightly regulated. Cellular copper uptake primarily occurs through transporter-mediated mechanisms. Dietary Cu^2+^ is reduced to Cu^+^ by STEAP metalloreductases before binding to SLC31A1, a plasma membrane transporter that facilitates cellular uptake of copper. Under low SLC31A1 activity, compensatory uptake occurs via SLC31A2 or SLC11A2. ZnT1, traditionally a zinc exporter, functions as a Cu^2+^/Zn^2+^ exchanger, importing copper while exporting zinc. Endocytosis represents an alternative pathway – CD44 mediates the internalization of Cu^2+^ in macrophages. Intracellular copper distribution relies on various chaperones. COX17 delivers copper to mitochondrial cytochrome c oxidase via SCO1/2, linking copper availability to oxidative phosphorylation. CCS activates SOD1 by copper insertion and transfers copper to MEK kinases in oncogenic signaling. ATOX1 transports copper to ATP7A/B transporters in the Golgi for subsequent distribution or efflux. Excess copper is sequestered by metallothioneins and glutathione, regulated by NRF2- or HIF-1α-mediated stress responses. ATP7A/B dynamically relocate to export copper via lysosomal exocytosis or plasma membrane trafficking. These transporters also confer chemoresistance in cancers through copper-dependent mechanisms and drug efflux. Copper metabolism integrates precise transport, compartmentalization, and export mechanisms to balance enzymatic needs with toxicity prevention

Copper is an essential trace element in living organisms, participating in numerous critical biological processes, such as redox reactions, electron transport, and serving as a cofactor for various enzymes. The precise regulation of copper ion metabolism is crucial for maintaining normal physiological functions at both cellular and organismal levels (Fig. 1). Dysregulation at any stage of copper metabolism can lead to a series of diseases, underscoring the importance of copper in maintaining cellular homeostasis and overall health [40–43]. For instance, excessive copper accumulation can result in oxidative stress and tissue damage, as seen in Wilson disease, while deficiency in copper distribution can impair enzyme function, leading to conditions such as Menkes disease. Furthermore, disruptions in copper homeostasis have been implicated in neurodegenerative disorders, cardiovascular diseases, and cancer progression.

### Cellular uptake of copper

#### Copper transporter and divalent metal transporter

The reduction of Cu^2+^ to Cu^+^ by cell-surface metalloreductases from the six-transmembrane epithelial antigen of the prostate (STEAP) family facilitates its cellular uptake. Copper transporter 1 (CTR1) encoded by solute carrier family 31 member 1 (*SLC31A1*) is a highly conserved transmembrane protein primarily localized to the plasma membrane, forming a homotrimeric complex. Extracellular Cu^2+^ must be reduced to Cu^+^ before binding to CTR1. The extracellular N-terminal domain of CTR1 is rich in methionine residues, which constitute copper-binding sites and selectively bind to Cu⁺. The transmembrane domain (TMD) forms a channel-like structure, allowing passive diffusion of Cu^+^ into the cytoplasm along the concentration gradient [44, 45]. In cases of *SLC31A1* deficiency [46], copper uptake can also occur through CTR2, which is encoded by *SLC31A2*, as a compensatory mechanism [47]. Cytosolic copper chaperones, such as copper chaperone for superoxide dismutase (CCS), antioxidant 1 copper chaperone (ATOX1), and cytochrome c oxidase copper chaperone COX17, rapidly capture Cu^+^ released from CTR1 via thiol groups, preventing free copper from inducing oxidative stress and delivering it to target proteins [40]. In addition, divalent metal transporter 1 (DMT1) encoded by *SLC11A2* is also involved in copper uptake [48]. The extracellular loop region of DMT1 preferentially binds to Fe^2+^, Zn^2+^, and Cu^2+^. Its copper affinity is significantly lower than that of CTR1, indicating that DMT1 primarily functions when CTR1 activity is limited or local copper concentrations are abnormally elevated [49].

#### Zinc transporter

Recently, zinc transporter 1 (ZnT1) has been found to be responsible for copper uptake [50]. ZnT1 is the first identified zinc transporter protein that is located to the plasma membrane and protects cells against zinc toxicity by mediating cytosolic zinc efflux [51–53]. By integrating genome-wide CRISPR screening, structural biology (cryo-EM), and in vivo models, Li et al. established ZnT1 as a pivotal Cu^2+^ transporter that is essential for cuproptosis and intestinal stem cell function [50]. Zn^2+^ and Cu^2+^ share the same binding site within ZnT1’s TMD. Cu^2+^ binding induces a conformational shift from outward-open to inward-open states, enabling cytosolic Cu^2+^ release. Simultaneously, ZnT1’s cytosolic histidine-rich loop recruits Zn^2+^ and delivers it to the TMD site during the conformational transition, leading to the efflux of Zn^2+^. Thus, ZnT1 functions as a Zn^2+^/Cu^2+^ exchanger, exporting Zn^2+^ and concomitantly importing Cu^2+^.

#### Endocytosis

Endocytosis is another important pathway for cells to uptake copper. Solier et al. reported that CD44 plays a central role in mediating the endocytosis of metal ions such as copper in activated monocyte-derived macrophages (aMDMs) [54, 55]. Activated macrophages not only highly express surface markers such as CD44 and CD86 but also exhibit significantly elevated intracellular level of copper. Knockdown of CD44 or the use of anti-CD44 antibodies inhibits copper uptake, while the expression levels of other copper transporters (e.g., CTR1, DMT1) remain unchanged. Hyaluronic acid specifically binds to Cu^2+^ via carboxyl groups to form complexes, which are internalized into cells via the CD44-mediated endocytic pathway. Finally, the lysosomal membrane copper transporter CTR2 is responsible for transporting released copper to the cytoplasm.

It was also found that the prion protein, which is abnormally up-regulated in *ATP7B*-deficient hepatocytes, promotes copper uptake through the endocytic pathway [56]. The prion protein binds to copper through its N-terminal histidine-rich region, which changes the conformation of the prion protein to facilitate its endocytic internalization and, thus, copper uptake [57–60]. After the copper-prion protein complex enters the cell, DMT1 and STEAP1 promote the release of copper from endosomal compartments to the cytoplasm. Animal models show that the knockout of *Prnp* gene, the prion protein-encoding gene in mice, can significantly improve the liver pathology of *Atp7b*-deficient mice and extend their survival. Interestingly, copper accumulation in *ATP7B*-deficient hepatic cells increases the expression of *PRNP* through the transcription factor MTF1 [61–63], thus forming a positive feedback loop that amplifies copper overload under the pathological condition.

However, the endocytic pathway can also prevent copper uptake. It was reported that CTR1 undergoes endocytosis from the plasma membrane when the copper level rises [64–66]. This serves as a safeguard mechanism against copper overload. The conserved His-Cys-His sequence at the invariant COOH-terminal of CTR1 is crucial in enabling this process. The internalization of CTR1 is clathrin- and dynamin-dependent and occurs through vesicles that contain Rab5 and EEA1. Conversely, when the cytosolic copper level declines, CTR1 can traffic back from the vesicles to the plasma membrane by binding to the retromer [67].

### Copper transport and distribution

Copper chaperones are specialized molecules responsible for the intracellular transport of copper. They ensure the precise delivery of copper to target proteins or subcellular compartments, preventing oxidative damage caused by free copper.

#### Cytochrome c oxidase copper chaperone COX17

COX17 is a core mitochondrial copper chaperone responsible for delivering copper to the cytochrome c oxidase (COX) assembly complex, which is a critical component of the mitochondrial respiratory chain. COX17 interacts with and transfers copper to the inner mitochondrial membrane proteins, synthesis of cytochrome c oxidase 1 (SCO1) and SCO2. In this way, it promotes the incorporation of copper into mitochondrially encoded cytochrome c oxidase subunit 2 (MT-CO2, also known as COX2) [68–70]. Mutations in SCO1/2 cause mitochondrial cardioencephalomyopathies, characterized by COX deficiency and energy metabolism collapse [71–73]. In addition, COX17 can also mediate the transfer of copper from the cytoplasm to MT-CO1 (also known as COX1) through the cytochrome c oxidase copper chaperone COX11 [74]. Therefore, the availability of copper is intricately associated with oxidative phosphorylation (OXPHOS) and robust mitochondrial function.

#### Copper chaperone for superoxide dismutase (CCS)

CCS is the specific co-factor for superoxide dismutase 1 (SOD1), and is responsible for inserting copper into the active site of SOD1 and promoting its proper folding and subcellular localization. SOD1 is a widely expressed free radical scavenger. It is a small protein with 154 amino acids that folds into an eight-stranded “Greek-key” β-barrel and binds to both copper and zinc. The copper is crucial for catalysis, while zinc plays a structural role [75, 76]. Misfolding of SOD1 is closely associated with amyotrophic lateral sclerosis (ALS). Dysfunction of CCS may lead to SOD1 aggregation, causing neuronal oxidative damage. Modulating the CCS-SOD1 interaction could be a potential therapeutic target for ALS [77, 78]. In addition, CCS can also transfer copper to the cuproenzymes MEK1 and MEK2. Both kinases act as components of the signaling pathway downstream of oncogenic RAS and RAF. The binding of copper to MEK1 and MEK2 is essential for their functionality, consequently promoting malignant transformation and drug resistance [79–81].

#### Antioxidant 1 copper chaperone (ATOX1)

Copper ions are transported to the trans-Golgi network via the antioxidant 1 copper chaperone (ATOX1), which binds to cytoplasmic Cu^+^ and delivers it to ATPase copper transporting alpha and beta (ATP7A and ATP7B) for subsequent distribution or efflux [82]. ATOX1 upregulation enhances genotoxic drug resistance and promotes cancer cell migration by copper-dependent activation of mediator of DNA damage checkpoint 1 (MDC1) and lysyl oxidase (LOX) proenzyme [83, 84]. Elevated intracellular copper triggers ATP7A/B relocation to lysosomes or melanosomes, facilitating copper excretion via exocytosis [85, 86].

Both CCS and ATOX1 can also mediate nuclear copper transport to modulate gene transcription. For instance, CCS and copper are essential for hypoxia-induced formation of the hypoxia-inducible factor 1α (HIF-1α)/E1A binding protein p300 complex in hepatocellular carcinoma [87], which likely inhibits cuproptosis [88], while ATOX1 acts as a copper-dependent transcription factor in endothelial and cancer cells [89].

### Copper functions

Copper plays a wide range of critical biological roles in mammalian physiological processes. As cofactors for numerous metabolic enzymes, they participate in various essential biochemical reactions. In cellular respiration, copper ions are key components of cytochrome c oxidase, located in the mitochondrial respiratory chain, where they are involved in electron transport and oxygen reduction processes [90–92]. This is crucial for maintaining mitochondrial function and cell survival [93, 94]. In antioxidant defense, copper ions serve as essential cofactors for SOD1 that converts highly reactive superoxide anions generated by the mitochondrial respiratory chain into less harmful peroxides and water, thus protecting cells from oxidative damage [95, 96]. SOD3, a copper-dependent enzyme, detoxifies oxygen radicals extracellularly in oxygen-rich tissues like lungs and blood vessels [95]. Loss of SOD3 is associated with vascular inflammation, pulmonary hypertension, and impaired angiogenesis [97, 98]. Secreted SOD3 shows therapeutic potential in reducing chemotherapy-induced oxidative stress [99]. Copper is also indispensable for the functional maturation and hemoglobin biosynthesis in the precursor state of red blood cells [100]. Additionally, copper contributes to the maintenance of the extracellular matrix. LOX and its related proteins utilize copper ions to catalyze the oxidative deamination of lysine residues, promoting the cross-linking of collagen and elastin [101–103]. This is vital for maintaining the normal structure and function of connective tissues and blood vessels [104]. Moreover, dopamine-β-hydroxylase utilizes copper ions to convert dopamine into norepinephrine, influencing the body’s neuroregulatory functions [105, 106]. Ceruloplasmin, with six copper atoms, oxidizes ferrous iron (Fe^2+^) to ferric iron (Fe^3+^), enabling iron transport by transferrin, which maintains iron homeostasis and modulates inflammation in hepatocytes, macrophages, and mammary glands [107–110]. Tyrosinase (TYR), a copper-containing enzyme, oxidizes tyrosine to melanin, protecting against UV damage [111]. Mutations in TYR cause albinism [112, 113], while temperature-sensitive variants lead to Siamese coloring patterns [114, 115].

In addition to serving as enzyme cofactors, copper also plays roles in structural stabilization, regulation, and signal transduction. Structurally, copper ions can bind to various intracellular proteins, stabilizing their structures. Examples include coagulation factor VIII [116], mucin MUC2 [117], and intracellular protein MEMO1 [118, 119]. Copper ions can also associate with the transcription factor SP1, which binds to GC-rich motifs of the gene promoters and participates in various cellular processes [120, 121]. Interestingly, SP1 modulates the expression of copper transporter genes via a regulatory loop [122–124]. Through allosteric regulation, copper ions stimulate the activity of ULK1 kinase [125] while inhibiting that of GSK3B kinase [126], indicating that they play roles in autophagy and neurological diseases. Additionally, copper ions mediate intercellular communication and signal transduction. In rat hippocampal neurons, copper ions are released in a calcium-dependent manner during potassium-induced depolarization [127]. Activation of NMDA receptors also leads to the release of copper ions into the synaptic cleft [128], although the molecular detail of this process remains unclear. This suggests a role for copper in signal transmission between neurons [129]. Moreover, intracellular copper ions can modulate receptor-mediated signal transduction by altering the activity of copper-dependent effectors. For example, copper inhibits the activity of cyclic AMP (cAMP)-degrading phosphodiesterase PDE3B, thereby increasing cAMP-dependent lipolysis [130]. Copper regulates circadian rhythms by modulating the suprachiasmatic clock and driving day-night expression of copper transporters like ATP7A [131], while circadian rhythms reciprocally control copper homeostasis through rhythmic induction of ATP7B in the pineal gland and retina [132, 133].

Both copper deficiency and excess cause significant effects on cellular functions. Copper deficiency, though rare, can lead to anemia, neutropenia, and neurological issues [134–136], likely due to impaired mitochondrial cytochrome c oxidase activity. Elevated copper disrupts mitochondrial function [137, 138], RNA processing [139–141], autophagy [142], and epigenetic regulation [54], while also impacting cytoskeleton dynamics and cell motility [143–145]. Notably, copper imbalance profoundly affects mitochondrial function—copper deficiency reduces ATP production and destabilizes complex IV [134], whereas excessive copper triggers oxidative damage, lipoylated protein aggregation, iron-sulfur (Fe-S) cluster loss, and impaired fusion-fission dynamics via a variety of mechanisms [38, 39, 146–148]. These findings highlight copper’s dual roles as both an essential cofactor and a potential toxicant, depending on its cellular levels.

### Copper sequestration and export

Copper homeostasis is tightly regulated through sequestration and export mechanisms to prevent cytotoxicity from labile copper ions. The sequestration primarily involves metallothioneins (MT1/MT2) and glutathione (GSH), while the export is mainly mediated by ATP7A and ATP7B.

#### Metallothioneins (MTs)

Metallothionein 1 (MT1) and MT2 are cysteine-rich proteins that bind to excess intracellular copper ions, limiting its cytotoxicity. Their expression is induced by NFE2 like bZIP transcription factor 2 (NFE2L2; also known as NRF2), a transcription factor activated by oxidative stress or copper overload and via KEAP1 inactivation [149, 150]. MTs also inhibit ATP7A trafficking from the trans-Golgi network, reducing copper export and promoting intracellular sequestration [151].

#### Glutathione (GSH)

Glutathione (GSH) is the primary non-protein thiol scavenger that chelates labile copper ions before MT binding. The synthesis of GSH is driven by NRF2-induced transcriptional upregulation of GCLM and GCLC [152]. The activation of NRF2 via KEAP1 mutations in various cancers, particularly non-small-cell lung cancer (NSCLC), enhances copper chelation and cytoprotection [153, 154]. In addition, HIF-1α-mediated induction of the cystine-glutamate transporter system x_c_^−^ (also known as xCT) and GCLM increases GSH synthesis, thus suppressing copper-dependent MEK1 signaling and promoting stemness in triple-negative breast cancer (TNBC) [155].

#### ATPase copper transporting alpha/beta (ATP7A/B)

ATP7A is widely expressed, while ATP7B regulates systemic copper in the liver [156]. Mutations disrupting ATP7A and ATP7B function result in the copper transport diseases Menkes disease and Wilson disease, respectively [157, 158]. These transporters localize to the trans-Golgi network under normal conditions but translocate to the plasma membrane during copper overload, expelling excess ions via ATP hydrolysis [159, 160]. Their activity is modulated by copper levels [85, 86, 161], metallothioneins [151], and KRAS and TFEB signaling pathways [162, 163]. KRAS-mediated ATP7A overexpression protects cancer cells from copper-induced cytotoxicity (possible cuproptosis) [162], while TFEB-induced ATP7B upregulation in ovarian cancer suggests that copper export is associated with lysosomal and autophagic pathways [163]. Both transporters also confer resistance to platinum-based chemotherapies in ovarian and liver cancers [163–165]. Their dual roles in copper-dependent and copper-independent (drug efflux) mechanisms highlight their complex contributions to tumorigenesis and therapy resistance.

## Regulation of cuproptosis

Cuproptosis is a copper-dependent form of RCD driven by the toxic accumulation of intracellular labile copper ions [38, 39]. It is mechanistically correlated with mitochondrial metabolism and protein aggregation, offering novel insights into the complex functions of copper as an essential micronutrient and a potent cytotoxic agent.

Tsvetkov and colleagues found that removing serum from the culture medium enhanced cellular resistance to the copper ionophore elesclomol because serum serves as the primary source of copper for cultured cells. Conversely, supplementing the medium with copper ions markedly increased cellular sensitivity to elesclomol. In addition, other copper ionophores, including disulfiram and NSC319726, were found to induce cell death in the presence of copper, whereas introducing other metals such as iron, cobalt, zinc, or nickel had no effect on this form of cell death. Copper chelators were used to confirm the role of copper in this process. Depleting the endogenous copper chelator GSH exacerbated elesclomol-induced cell death, whereas adding the exogenous chelator tetrathiomolybdate (TTM) suppressed it. Further experiments revealed that inhibitors targeting apoptosis, ferroptosis, necroptosis, or oxidative stress failed to rescue cells from copper-induced death. These findings collectively indicate that cuproptosis represents a distinct mechanism from other established cell death pathways.

Cells reliant on mitochondrial metabolism exhibited increased sensitivity to copper ionophores compared to those primarily utilizing glycolysis [38, 39]. Consistent with this, blocking complexes I and II of the electron transport chain (ETC) or mitochondrial pyruvate uptake alleviated cuproptosis. Additionally, cells cultured under hypoxic conditions resisted cuproptosis; however, artificially activating the HIF pathway under normoxia did not alter the susceptibility. This finding further emphasizes the critical role of mitochondrial respiration in cuproptosis. Notably, the mitochondrial uncoupling agent FCCP, which diminishes mitochondrial membrane potential and ATP production, did not influence cuproptosis, implying that ATP synthesis is dispensable for this process. Furthermore, elesclomol significantly reduced the spare respiratory capacity of mitochondria but left basal and ATP-linked respiration unaffected, suggesting that the copper may target TCA cycle components directly, without disrupting ETC activity or ATP generation.

To unravel the molecular basis of cuproptosis, they conducted a genome-wide CRISPR-Cas9 screen [39]. Ten genes were identified as important regulators of this process, including *ferredoxin 1* (*FDX1*), *lipoic acid synthetase* (*LIAS*), *lipoyltransferase 1* (*LIPT1*), *dihydrolipoamide dehydrogenase* (*DLD*), *DLAT*, *pyruvate dehydrogenase E1 subunit alpha 1* (*PDHA1*), *pyruvate dehydrogenase E1 subunit beta* (*PDHB*), *metal regulatory transcription factor 1* (*MTF1*), *glutaminase* (*GLS*), and *cyclin dependent kinase inhibitor 2 A* (*CDKN2A*). Among these, *FDX1* encodes a reductase that reduces Cu^2+^ to the more cytotoxic Cu^+^; *LIPT1*, *LIAS*, and *DLD* are involved in the lipoic acid biosynthesis pathway; and *DLAT*, *PDHA1*, and *PDHB* encode subunits of the pyruvate dehydrogenase (PDH) complex, highlighting the importance of copper, lipoylation, and mitochondrial pyruvate in cuproptosis. Interestingly, *MTF1*, *GLS*, and *CDKN2A* negatively correlated with cuproptosis, indicating that copper homeostasis, glutamine utilization, and possibly cell cycle progression play roles in cuproptosis. Copper was observed to directly bind to lipoylated TCA cycle proteins, DLAT and dihydrolipoamide S-succinyltransferase (DLST), inducing their aggregation via disulfide bonds and promoting the degradation of Fe-S cluster proteins. Crucially, FDX1 emerged as a central regulator, as its depletion abolished protein lipoylation, suppressed cellular respiration, caused pyruvate and α-ketoglutarate accumulation, reduced succinate levels, and stabilized Fe-S cluster proteins. These results demonstrate that excessive copper triggers FDX1-dependent aggregation of lipoylated TCA cycle proteins and destabilization of Fe-S cluster proteins, consequently leading to cuproptosis.

Finally, the study investigated how intracellular copper levels influence cuproptosis by modulating the copper importer CTR1 and exporter ATP7B in cellular and animal models [39]. Overexpression of CTR1 heightened cellular sensitivity to CuCl_2_-induced aggregation of lipoylated proteins and Fe-S cluster degradation. This sensitivity was partially reversed by copper chelators or by knocking down FDX1 or LIAS. These findings were corroborated in a Wilson disease mouse model (*Atp7b*^*−/−*^), where livers of *Atp7b*^*−/−*^ mice exhibited pronounced loss of lipoylated and Fe-S cluster proteins compared to *Atp7b*^*+/−*^ and wild-type mice, confirming that intracellular copper accumulation drives cuproptosis in vivo.

The studies by Tsvetkov et al. [38, 39] elucidate the mechanism of cuproptosis and underscore the significance of copper homeostasis in diseases such as cancer. Nevertheless, critical questions remain unresolved in this nascent field. Is cuproptosis modulated by cellular stress signals? How do metabolic pathways, including glycolysis, OXPHOS, and copper homeostasis, interplay with cuproptosis in cancer? Could therapeutic strategies triggering cuproptosis be developed for cancer treatment?

### Regulation of cuproptosis by p53

The tumor suppressor p53 maintains genomic stability and thus prevents tumorigenesis by regulating cell cycle arrest, DNA repair, and apoptosis in response to various cellular stresses [166]. It also orchestrates diverse cell death pathways, including necroptosis, pyroptosis, ferroptosis, and autophagic cell death, mostly through transcriptional regulation of gene expression [167–170]. While cuproptosis has been identified as a mitochondrial metabolism-dependent death mechanism since 2019 [38], its intersection with p53 signaling remained unexplored. The study by Liao et al. elucidated a novel p53-dependent regulatory axis governing cuproptosis sensitivity in colorectal and breast cancers through the circular RNA circFRMD4A, revealing critical metabolic rewiring mechanisms and therapeutic implications [171].

Expression microarray and quantitative PCR analysis identified circFRMD4A as a p53-regulated circular RNA that is induced by DNA damage stress (5-FU/cisplatin) and MDM2 inhibitors (nutlin-3 and APG-115) in wild-type p53 (wtp53)-harboring cancer cells. p53 transcriptionally activates the expression of the host gene *FRMD4A* by binding to a p53-responsive element on its promoter. Subsequently, the RNA-binding protein EWSR1 facilitates the formation of circFRMD4A through intronic Alu element interactions. Clinical analysis revealed that circFRMD4A downregulation in colorectal and breast tumors correlated with advanced T stage, lymph node metastasis, and poor survival of patients. Consistently, ectopic circFRMD4A suppressed cancer cell proliferation, colony formation, migration, and xenograft tumor growth. In contrast, the depletion of circFRMD4A promoted the progression of colorectal and breast cancers. The authors further found that circFRMD4A overexpression enhanced cell sensitivity to elesclomol-induced cuproptosis, while its knockdown conferred cuproptosis resistance in cancer cells. Mechanistically, circFRMD4A binds to pyruvate kinase M2 (PKM2) via its A1/B domains, thereby inhibiting the tetramerization of PKM2 and thus its enzymatic activity. This inactivation of PKM2 redirects glycolytic flux from lactate production to the TCA cycle and mitochondrial metabolism, consequently enhancing the sensitivity to cuproptosis in colorectal and breast cancer cells.

Although both p53 agonists and genotoxic agents can induce p53 stabilization and activation, the authors reported that nutlin-3 and APG-115, but not cisplatin or 5-FU, synergized with elesclomol to suppress the growth of colorectal and breast cancer cells. This is partially because DNA damage stress may lead to mitochondrial dysfunction [172, 173]. Specifically, moderate DNA damage stress triggers mitophagy to remove damaged and depolarized mitochondria, while drastic stress is able to induce mitochondrial outer membrane permeabilization and apoptosis [174]. The impairment of mitochondrial integrity and function may lead to insensitivity to cuproptosis [171]. Another interesting observation is that copper and cisplatin share the common transport mechanism involving the copper transporter CTR1 [175, 176], suggesting that they have to compete for the entry into cells.

This study for the first time unveils the molecular mechanism by which p53 regulates the glycolytic flux through circFRMD4A and enhances the sensitivity to cuproptosis, and provides a clinically translatable strategy—p53 activation combined with copper toxicity – for treating wtp53-harboring malignancies.

In addition to the circFRMD4A-PKM2 axis, p53 may also regulate cuproptosis through multiple mechanisms, as proposed by Xiong et al. [170]. First, p53 inhibits glycolysis via the repression of glucose transporters (GLUTs) [177–179], glycolytic enzymes such as hexokinase 2 (HK2), phosphofructokinase (PFK1), and lactate dehydrogenase A (LDHA) [180–183], and HIF-1α [184], which may lead to a compensate metabolic shift diverting pyruvate into mitochondria, fueling the TCA cycle. Conversely, mutant p53 (mtp53) promotes glycolysis through HK2 upregulation [185] and mTOR-PKM2 activation [186, 187], potentially conferring cuproptosis resistance. Second, p53 amplifies mitochondrial oxidative capacity through multiple pathways. p53 induces glutaminase (GLS2) [188, 189] and aspartate transporter solute carrier family 1 member 3 (SLC1A3) [190], providing substrate supply for α-ketoglutarate and citrate synthesis. p53 also upregulates SCO2 (critical for cytochrome c oxidase assembly) [191] and apoptosis inducing factor mitochondria associated 1 (AIFM1; required for complex I stability) [192], bolstering ETC efficiency. Furthermore, p53 promotes fatty acid oxidation while suppressing lipogenesis through multiple mechanisms [193–196], thereby increasing acetyl-CoA pools for mitochondrial energetics. These findings suggest that p53 may promote cuproptosis sensitivity by supporting mitochondrial respiration. Third, Fe-S clusters, essential for ETC and DNA repair enzymes, are destabilized during cuproptosis. p53 enhances Fe-S biogenesis by upregulating scaffold proteins, such as Fe-S cluster assembly enzyme (ISCU) [197] and frataxin (also known as FXN) [198, 199], and ferredoxin reductase (FDXR) [200–202]. However, p53 also represses heat shock protein family A (Hsp70) member 9 (HSPA9) [203], possibly leading to the perturbation of Fe-S cluster biogenesis. These findings suggest that p53 may regulate cuproptosis by coordinating the biogenesis and homeostasis of Fe-S clusters. Finally, p53 may promote or inhibit cuproptosis through the regulation of GSH biogenesis. p53 represses the expression of solute carrier family 7 member 11 (SLC7A11) [204], a component of the cystine-glutamate transporter system x_c_^−^, which possibly leads to the depletion of GSH and the increase in labile copper ions. In contrast, p53 also activates TIGAR (TP53 induced glycolysis regulatory phosphatase) [181] or NRF2 [205], possibly mitigating copper toxicity. This duality allows p53 to act as a sensor, eliminating copper-overloaded cells while protecting stressed cells with reversible damage. For more comprehensive perspectives on the potential regulatory roles of p53 in cuproptosis, we direct readers to the seminal review article by Xiong et al. [170].

**Fig. 2:**
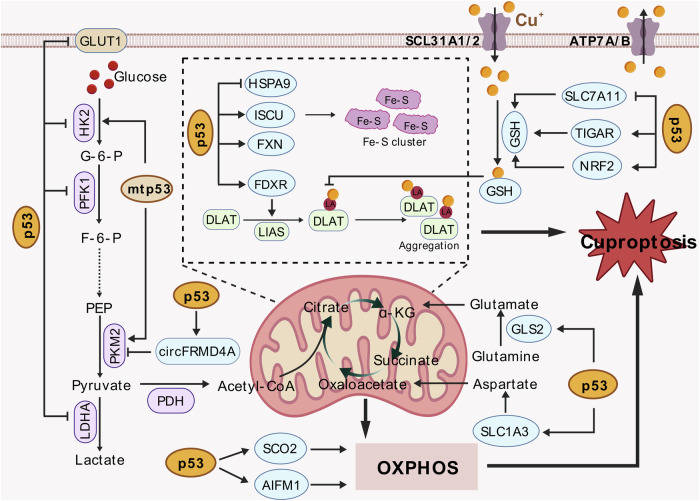
Regulation of cuproptosis by p53. p53 regulates cuproptosis possibly through multiple mechanisms. A recent study reveals that p53 enhances cuproptosis sensitivity in cancers via the circular RNA circFRMD4A. It binds to PKM2, blocking its tetramerization and glycolytic activity, thereby redirecting glucose flux to the TCA cycle to amplify mitochondrial respiration – a prerequisite for copper-induced toxicity. Beyond the experimentally validated circFRMD4A-PKM2 axis, p53 is proposed to regulate cuproptosis through different pathways. p53 suppresses glycolysis by inhibiting GLUTs and glycolytic enzymes (HK2, PFK1, LDHA), providing a key metabolic environment for sensitizing cuproptosis. Conversely, mutant p53 promotes glycolysis by activating HK2 and PKM2, potentially inducing cuproptosis resistance. p53 also amplifies mitochondrial oxidative capacity by upregulating GLS2, SLC1A3, SCO2, and AIFM1. Additionally, p53 modulates Fe-S cluster biogenesis via ISCU, FXN, FDXR, and HSPA9, which balances Fe-S stability and affects the lipoylation pathway. In redox regulation, p53 reduces GSH by suppressing SLC7A11 but activates TIGAR or NRF2 to mitigate copper toxicity, acting as a dual sensor to eliminate copper-overloaded cells or protect stressed cells with reversible damage

Collectively, p53 emerges as a central regulator of cuproptosis susceptibility, primarily through metabolic reprogramming. The elucidated circFRMD4A-PKM2 axis demonstrates how p53 activation directs glycolytic flux toward mitochondrial respiration, thereby creating a metabolic context favorable for copper-induced cell death. This positions p53 not only as a genomic guardian but also as a key metabolic determinant of cuproptosis, offering a rationale for combining p53 agonists with copper-based therapies in wtp53-harboring malignancies (Fig. 2).

### Regulation of cuproptosis by HIF-1α

**Fig. 3:**
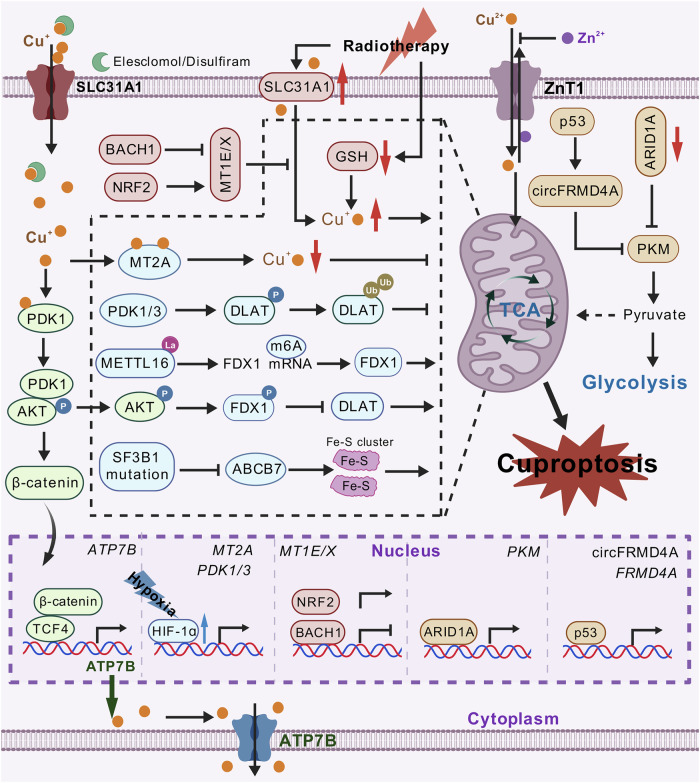
Regulation of cuproptosis via diverse pathways. Hypoxia-driven HIF-1α induces cuproptosis evasion by degrading DLAT via PDK1/3 and upregulating metallothionein MT2A to chelate mitochondrial copper. The Wnt/β-catenin pathway, activated by copper-PDK1 binding, promotes ATP7B-mediated copper efflux in cancer stem cells. AKT1 phosphorylates FDX1, which disrupts DLAT lipoylation and enables glycolytic reprogramming, thereby triggering cuproptosis resistance. METTL16, regulated by lactylation via AARS1/2 and delactylation via SIRT2, stabilizes FDX1 mRNA via m6A methylation, linking lactate metabolism to cuproptosis sensitivity. SF3B1-mutant acute myeloid leukemia shows vulnerability to copper overload via Fe-S cluster defects caused by ABCB7 mis-splicing and downregulation. p53 activation or ARID1A-deficiency redirects glycolytic flux to the TCA cycle by inhibiting PKM2. ZnT1, traditionally a zinc exporter, acts as a critical Cu^2+^ importer, which is essential for the susceptibility to copper-induced cell death. Radiotherapy also induces cuproptosis through SLC31A1 upregulation and GSH depletion but faces resistance via BACH1/NRF2-mediated MT1E/X expression

Beyond p53 regulation, cuproptosis is orchestrated by a network of signaling molecules (Fig. 3). While cuproptosis represents a promising anticancer strategy, its efficacy is limited in solid tumors due to their hypoxic tumor microenvironment (TME) – a hallmark feature associated with therapy resistance [39, 206]. Under hypoxia, cancer cells shift from OXPHOS to glycolysis (the “Warburg effect”) to sustain energy production and redox homeostasis, a transition regulated by the transcription factor HIF-1α [184, 207, 208]. Through spatial transcriptomics and single-cell RNA sequencing across multiple cancer types, Yang et al. discovered a striking inverse correlation between hypoxia signatures and cuproptosis susceptibility [88]. Hypoxic tumor regions exhibited reduced expression of cuproptosis markers like DLAT and FDX1, as well as diminished mitochondrial copper accumulation. Mechanistically, HIF-1α triggers a dual-axis resistance program. First, hypoxia stabilizes HIF-1α, which transcriptionally upregulates pyruvate dehydrogenase kinases PDK1/3. These kinases phosphorylate DLAT at Ser100, promoting its ubiquitin-mediated degradation. As DLAT is essential for copper-induced lipoylated protein aggregation, its loss desensitizes cells to cuproptosis. CRISPR-mediated PDK1/3 knockout restored DLAT expression and cuproptosis sensitivity, further validating this regulatory axis. Second, HIF-1α induces metallothionein-2A (MT2A) expression by direct binding to the hypoxia response element on its promoter. MT2A is a cysteine-rich metal-binding protein that chelates mitochondrial copper while leaving total cellular copper unaffected. MT2A depletion increased mitochondrial copper accumulation and restored cuproptosis sensitivity even under hypoxia. Clinically, high MT2A expression correlated with advanced tumor stages and poor survival across TCGA cohorts.

Interestingly, sublethal copper doses stabilized HIF-1α by inhibiting VHL-mediated ubiquitination and degradation. This copper-HIF-1α axis upregulated glycolytic enzymes, such as GLUT1 and LDHA, to compensate for TCA cycle inhibition, providing a metabolic buffer against cuproptosis. These findings lead to several potential combination strategies. GLUT1 inhibition by BAY-876 synergized with copper ionophores to enhance cell death. More importantly, combining the HIF-1α inhibitor PX-478 with low-dose elesclomol-Cu achieved tumor suppression comparable to high-dose elesclomol-Cu while avoiding hepatotoxicity.

This work resolves two longstanding questions in copper biology: firstly, how hypoxic tumors evade copper toxicity, and secondly, why cuproptosis efficacy varies across metabolic contexts. By identifying HIF-1α as a central regulator of both copper metabolism (via MT2A) and mitochondrial vulnerability (via PDK1/3-DLAT), the authors provide a mechanistic basis for targeting the hypoxia-cuproptosis axis. The combination of HIF-1α inhibitors with copper ionophores addresses critical limitations of metal-based therapies, such as their narrow therapeutic windows and off-target toxicity. Given the prevalence of hypoxia in solid tumors, this combination strategy could broaden the applicability of cuproptosis in precision oncology. These findings redefine copper homeostasis as a dynamic interface between environmental stress and cell fate, offering novel biomarkers (e.g., DLAT phosphorylation and MT2A levels) and therapeutic combinations to exploit metal toxicity in cancer treatment.

In summary, HIF-1α orchestrates a multifaceted defense mechanism against cuproptosis under hypoxia. This work provides a clear mechanistic explanation for the limited efficacy of cuproptosis induction in hypoxic solid tumors and establishes the HIF-1α pathway as a promising target to broaden the application of copper ionophores in cancer therapy.

### Regulation of cuproptosis by Wnt/β-catenin

The Wnt/β-catenin pathway is a highly conserved signaling cascade that regulates critical processes such as cell fate determination, proliferation, and tissue homeostasis [209, 210]. It operates by stabilizing β-catenin when Wnt ligands bind to Frizzled receptors, allowing β-catenin to accumulate and translocate into the nucleus to activate target genes through T-cell factor/lymphoid enhancer factor (TCF/LEF). It is frequently hyperactivated to drive stemness and therapy resistance in cancers. Recently, Liu et al. investigated how Wnt/β-catenin signaling modulates copper homeostasis to confer cuproptosis resistance in cancer stem cells (CSCs) [211].

Transcriptomic profiling of head and neck squamous cell carcinoma (HNSCC) cells treated with elesclomol-Cu revealed robust activation of the Wnt/β-catenin pathway, marked by nuclear β-catenin accumulation and phosphorylation of AKT (Thr308)/GSK3β (Ser9). These effects were reversible upon copper chelation, indicating a correlation between cuproptosis and the Wnt signaling pathway. In vivo, elesclomol-Cu suppressed tumor growth but paradoxically upregulated CSC markers, such as CD44 and CD133, and β-catenin, suggesting an adaptive activation of the pathway during cuproptosis. Mechanistically, intracellular copper that is transported by CTR1 binds to PDK1, a key kinase in the PI3K/AKT pathway. Structural and biophysical analyses demonstrated high-affinity copper binding to PDK1, enhancing its interaction with AKT and activating downstream β-catenin signaling. Mutation of PDK1’s copper-binding residues (H117A/H203A) abolished this activation, confirming copper-PDK1 binding as a central mechanism to activate the Wnt pathway during cuproptosis.

The Wnt/β-catenin-transcription factor 4 (TCF4) axis further reinforced cuproptosis resistance through transcriptional induction of the copper exporter ATP7B, which was validated through chromatin profiling and functional assays. CSCs, characterized by elevated TCF4 and ATP7B expression, exhibited reduced labile copper pools and increased resistance to cuproptosis. Genetic or pharmacological inhibition of TCF4 sensitized CSCs to cuproptosis by depleting ATP7B, elevating intracellular copper, and reducing GSH buffering capacity. Strikingly, ATP7B overexpression rescued TCF4-deficient cells from cuproptosis by restoring copper efflux and suppressing DLAT oligomerization.

Therapeutic targeting of this axis demonstrated promising efficacy. In HNSCC xenografts, combining LF3, a disruptor of β-catenin/TCF4 interaction, with elesclomol-Cu synergistically reduced tumor growth compared to monotherapies. In immunocompetent B16-F10 melanoma models, this combination enhanced CD8^+^ T-cell infiltration, overcoming Wnt-mediated immunosuppression. These findings indicate the dual role of Wnt inhibition, which potentiates cuproptosis and reprograms the tumor microenvironment. Clinically, CTR1 and TCF4 emerged as prognostic biomarkers, as their overexpression correlated with advanced stage, metastasis, and poor survival in HNSCC. Their association with Wnt pathway activity and CSC properties positions them as predictors of cuproptosis sensitivity.

This work elucidates a paradigm wherein Wnt/β-catenin signaling enables CSCs to bypass copper toxicity through ATP7B-mediated efflux. By delineating the copper-PDK1-AKT/β-catenin-TCF4-ATP7B axis, the study provides a mechanistic foundation for combining Wnt inhibitors with copper ionophores to target therapy-resistant malignancies.

### Regulation of cuproptosis by AKT

Although recent discoveries have highlighted cuproptosis as a promising therapeutic approach, elevated copper levels are associated with tumor progression and poor prognosis in cancers such as triple-negative breast cancer (TNBC). The mechanisms underlying copper’s conflicting effects – supporting cell proliferation while also inducing cell death – remain incompletely understood. TNBC cells exhibit resistance to cuproptosis despite high copper levels, thus prompting a study to investigate the regulatory pathways involved in this resistance and explore therapeutic strategies to overcome it [212].

Functional assays confirmed that TNBC cells exhibited reduced sensitivity to cuproptosis, as evidenced by diminished lipoylation and oligomerization of DLAT proteins. This resistance suggested the presence of intrinsic regulatory mechanisms that counteract copper-induced cell death in TNBC. To identify these mechanisms, the researchers generated cuproptosis-resistant TNBC cell lines and performed phosphoproteomic profiling. This analysis revealed significant enrichment of phosphorylation events in pathways related to insulin signaling and AGC kinase activity, with AKT1 emerging as a central player. AKT1, a serine/threonine kinase frequently hyperactivated in cancers, was found to be phosphorylated in response to elesclomol-Cu treatment. Further experiments demonstrated that elesclomol-Cu enhanced the interaction between AKT1 and PDK1, a kinase responsible for AKT1 activation, leading to sustained AKT1 signaling in TNBC cells, which was consistent with the study mentioned above [211]. Crucially, genetic or pharmacological inhibition of AKT1 sensitized TNBC cells to cuproptosis, restoring DLAT lipoylation and oligomerization. These findings position AKT1 as a critical regulator of cuproptosis resistance in TNBC.

FDX1 was further identified as a direct substrate of AKT1. AKT1 phosphorylates FDX1 at Ser63, a modification that disrupts FDX1’s ability to promote lipoylation of DLAT and other TCA cycle enzymes. Specifically, phosphorylation of FDX1 at Ser63 impaired its interaction with DLAT, leading to defective protein lipoylation and subsequent resistance to copper-induced proteotoxic stress. These results establish a pivotal link between AKT1 activity, FDX1 phosphorylation, and cuproptosis resistance in TNBC.

Besides its role in cuproptosis, AKT1-mediated FDX1 phosphorylation was shown to drive metabolic reprogramming in TNBC cells. Phosphorylated FDX1 promoted glycolysis, as evidenced by increased pyruvate and lactate production, enhanced extracellular acidification rates (ECAR), and upregulation of glycolytic enzymes such as GLUT1, HK2, PKM2, and LDHA. It also suppressed mitochondrial respiration, leading to reduced oxygen consumption rates (OCR) and ATP production via OXPHOS. This metabolic shift toward glycolysis enabled TNBC cells to sustain rapid proliferation despite mitochondrial dysfunction. The adaptation of TNBC cells to glycolysis can also contribute to cuproptosis resistance.

The translational implications of these findings were explored through preclinical testing of combination therapy targeting both AKT1 and copper metabolism. The AKT1 inhibitor MK2206 combined with elesclomol synergistically reduced TNBC cell viability and colony formation in vitro. In mouse xenograft models, this combination therapy significantly inhibited tumor growth compared to monotherapy, accompanied by increased DLAT lipoylation in tumor tissues.

Therefore, AKT1-mediated phosphorylation of FDX1 represents a direct post-translational mechanism of cuproptosis resistance. This pathway not only inactivates a key driver of copper toxicity but also reinforces a glycolytic phenotype that further diminishes mitochondrial susceptibility. Since AKT is frequently hyperactive in TNBC, this mechanism explains the paradox of high copper levels coexisting with cuproptosis resistance, and underscores AKT inhibition as a potent sensitizer to copper-based therapies.

### Regulation of cuproptosis by METTL16

Cuproptosis is governed by a critical axis involving alanyl-tRNA synthetase (AARS)/ sirtuin 2 (SIRT2)-mediated lactylation of methyltransferase 16, RNA N6-adenosine (METTL16), and subsequent METTL16-dependent m6A methylation of FDX1 mRNA in gastric cancer (GC) [213]. Analysis of 48 paired GC and adjacent tissues revealed significantly higher copper levels in tumors, particularly in advanced-stage and mucinous adenocarcinomas—a rare, aggressive GC subtype associated with poor prognosis. Elevated copper correlated with worse overall survival (OS), disease-free survival (DFS), and markers of proliferation (Ki-67) and inflammation (neutrophil/lymphocyte ratio). Intriguingly, mucinous adenocarcinomas exhibited higher copper levels than non-mucinous subtypes, aligning with their resistance to conventional therapies. These clinical observations suggested that leveraging copper’s cytotoxic potential could offer a therapeutic avenue for refractory GC.

While FDX1 protein levels were elevated in GC tissues, the mechanisms regulating its expression under copper stress remained unclear. The study identified METTL16, an m6A methyltransferase, as a key mediator. METTL16 knockdown or knockout in GC cells conferred resistance to copper ionophores (elesclomol/disulfiram), whereas METTL16 overexpression sensitized cells to cuproptosis. Mechanistically, METTL16 deposited m6A modifications on FDX1 mRNA, enhancing its stability and translation. Clinically, METTL16 and FDX1 levels were positively correlated in GC tissues, suggesting their functional association.

The study further uncovered a novel regulatory layer involving lactate-derived lactylation of METTL16. Copper stress, despite not altering METTL16 expression, induced lactylation at Lys229, a modification detected with mass spectrometry and validated using a site-specific antibody. Lactylation at Lys229 enhanced METTL16’s methyltransferase activity by destabilizing its autoinhibitory conformation. This modification was catalyzed by aminoacyl-tRNA synthetases AARS1/AARS2, which interact with METTL16 upon copper treatment. Conversely, the NAD^+^-dependent deacetylase SIRT2 acted as a delactylase, removing lactylation marks from METTL16 and suppressing its activity. SIRT2 overexpression reduced FDX1 m6A levels and protein expression, whereas SIRT2 inhibition via knockdown or its inhibitor AGK2 increased METTL16 lactylation and FDX1-driven cuproptosis.

The translational implications of this axis were tested in preclinical models. Reconstitution with lactylation-mimetic METTL16-K229E restored FDX1 expression and cuproptosis sensitivity in METTL16-knockdown GC cells. In xenograft models, tumors derived from METTL16-K229E cells showed significant growth inhibition upon elesclomol treatment, accompanied by elevated FDX1 and DLAT lipoylation. Combining elesclomol with the SIRT2 inhibitor AGK2 synergistically induced cuproptosis in GC cells and suppressed tumor growth in mice, suggesting that targeting both copper delivery and METTL16 lactylation could be a potential therapeutic strategy.

The interplay between copper and lactate metabolism has significant implications. Tumors with high glycolytic activity produce abundant lactate that may facilitate cuproptosis via METTL16 lactylation when copper levels rise. This metabolic vulnerability could be exploited in cancers with dual reliance on glycolysis and OXPHOS, such as certain GC subtypes.

### Regulation of cuproptosis by ARID1A

Hepatocellular carcinoma (HCC) is the most common primary liver cancer. Despite advances in immunotherapy and targeted therapies, treatment resistance and recurrence persist due to the genetic heterogeneity of HCC and the scarcity of actionable driver mutations. A significant proportion of HCC tumors harbor loss-of-function mutations in AT-rich interaction domain 1 A (ARID1A), a key subunit of the SWI/SNF chromatin remodeling complex involved in transcriptional regulation and chromatin accessibility. While synthetic lethality strategies targeting *ARID1A*-deficient cancers have shown promise in other cancer types, their application in HCC remains underexplored. Xing et al. uncovered a metabolic vulnerability in *ARID1A*-deficient HCC, revealing a novel therapeutic avenue through cuproptosis [214].

A genome-wide CRISPR-Cas9 knockout screen was conducted in *ARID1A*-KO HCC cells. This screen identified synthetic lethal interactions with genes critical to the TCA cycle and OXPHOS. Key TCA cycle enzymes, including aconitase 2 (ACO2), succinate dehydrogenase complex flavoprotein subunit A (SDHA), and fumarate hydratase (FH), emerged as top hits, suggesting that ARID1A loss forces HCC cells to rely on mitochondrial respiration for survival. Metabolic analysis further revealed a dramatic shift from glycolysis to OXPHOS in *ARID1A*-deficient cells. Mechanistically, ARID1A directly binds to the PKM promoter, where it maintains chromatin accessibility for transcriptional activation. Loss of *ARID1A* reduced PKM expression and activity, impairing pyruvate production and forcing cells to depend on mitochondrial respiration.

In the cancer cell-derived xenograft (CDX) model, elesclomol selectively inhibited tumor growth in *ARID1A*-KO HCC, as well as reduced the levels of proliferation marker Ki-67 and TCA cycle proteins, such as SDHA and FDX1. HCC patient-derived xenografts (PDXs) with the *ARID1A* nonsense mutation (p.E193*) mirrored these results, with significant tumor regression and no overt toxicity.

The synthetic lethal interaction between *ARID1A* deficiency and TCA cycle dependency emphasizes metabolic reprogramming as a therapeutic Achilles’ heel. *ARID1A* status could serve as a biomarker to stratify HCC patients for copper-based therapies, providing a precision medicine approach for a genetically defined subgroup.

### Regulation of cuproptosis by SF3B1

Acute myeloid leukemia (AML), a heterogeneous hematologic malignancy with poor outcomes in high-risk subgroups, remains a therapeutic challenge due to limited targeted strategies. Among recurrent genetic lesions in AML, mutations in *splicing factor 3b subunit 1* (*SF3B1*)—a core component of the spliceosome—are associated with aberrant mRNA splicing, mitochondrial dysfunction, and adverse prognosis. Moison et al. conducted a high-throughput screen of 10,000 compounds across 56 primary AML specimens. The screen identified S767, a benzenesulfonamide derivative, as selectively toxic to poor-prognosis AML subtypes, particularly those with *SF3B1* mutations and complex karyotypes [215].

S767 disrupts intracellular metal homeostasis by depleting iron and elevating copper and zinc. CRISPR-Cas9 screen revealed the biosynthesis and transport of Fe-S cluster as critical determinants of S767 sensitivity. Synthetic lethality was observed with loss of Fe-S cluster assembly genes, such as NFS1 cysteine desulfurase and HSPA9, and the Fe-S cluster transporter ATP binding cassette subfamily B member 7 (ABCB7), while the abrogation of mitochondrial Fe-S cluster-loading factors such as glutaredoxin 5 (GLRX5) and iron-sulfur cluster assembly factor IBA57 conferred resistance. Remarkably, *SF3B1* mutations were found to drive ABCB7 mis-splicing and downregulation, mirroring the Fe-S cluster-deficient phenotype. In addition, UM4118, a nanomolar-active analog of S767, was identified as exhibiting copper-specific ionophore activity. UM4118 impaired mitochondrial respiration, reducing maximal oxygen consumption and inducing DLAT aggregation.

This study positions *SF3B1* mutations as biomarkers for copper ionophore therapy, offering a tailored approach for adverse-risk AML. The synthetic lethality between Fe-S cluster deficiency and copper overload highlights mitochondrial metabolism as an exploitable vulnerability, particularly in malignancies with spliceosomal defects. UM4118, with its copper selectivity and nanomolar potency, could represent an optimized copper ionophore and serve as a promising therapeutic candidate for precision oncology.

### Regulation of cuproptosis by ZnT1

Although copper homeostasis is classically regulated by transporters such as CTR1 (Cu^+^ importer) and ATP7A/B (Cu^+^ exporters), the mechanisms underlying Cu^2+^ transport and its role in cuproptosis remain poorly understood. Li et al. identified solute carrier family 30 member 1 (SLC30A1; also known as ZnT1), a member of the zinc transporter (ZnT) family traditionally associated with Zn^2+^ efflux, as a critical Cu^2+^ transporter that drives cuproptosis and maintains intestinal Cu-Zn homeostasis [50].

Using a genome-wide CRISPR-Cas9 screen in HeLa cells treated with CuSO_4_, ZnT1 emerged as the top gene whose knockout conferred resistance to Cu²⁺-induced cuproptosis. Surprisingly, despite ZnT1’s known role in Zn^2+^ export, its Cu^2+^ transport activity was distinct from other ZnT family members (e.g., ZnT10) and independent of CTR1 that primarily mediates Cu^+^ uptake. Cryo-EM structures of ZnT1 in the apo and Zn^2+^-bound states demonstrated a homodimeric architecture stabilized by a unique inter-subunit disulfide bond in its extracellular cysteine-rich loop, a feature absent in other ZnTs. ZnT1’s TMD harbored a conserved metal-binding site (H43, D47, H251, D255) shared by both Cu^2+^ and Zn^2+^, with extracellular Zn^2+^ competitively inhibiting Cu²⁺ transport.

ZnT1’s dual roles in Cu^2+^ import and Zn^2+^ export were physiologically validated in intestinal epithelial-specific ZnT1-KO mice. These mice exhibited copper deficiency in enterocytes, loss of Lgr5^+^ stem cells, and reduced proliferation of transit-amplifying cells – phenotypes exacerbated under low-copper diets. While ZnCl_2_ supplementation rescued systemic zinc deficiency and lethality, it failed to restore intestinal stem cell defects, demonstrating ZnT1’s non-redundant role in Cu^2+^ uptake for epithelial maintenance.

The identification of ZnT1 as a major Cu^2+^ importer redefines our understanding of cellular copper uptake. Its role as a Zn^2+^/Cu^2+^ exchanger explains the physiological basis of zinc acetate in treating Wilson disease (copper overload), as Zn^2+^ competes with Cu^2+^ for ZnT1 binding, thereby inhibiting pathological copper accumulation. Future studies targeting ZnT1’s Cu^2+^-binding site or disulfide bond may yield precision therapies for cuproptosis-related pathologies or copper dysregulation syndromes.

### Regulation of cuproptosis by radiotherapy

Radiotherapy is a cornerstone of cancer treatment, exerting its antitumor effects through DNA damage and reactive oxygen species (ROS) generation. However, radioresistance, which is driven by evasion of RCD pathways, remains a major clinical challenge. Lei et al. established cuproptosis as a critical mediator of radiotherapy-induced tumor suppression and identified its dysregulation as a key driver of radioresistance [216]. This study demonstrated that radiotherapy induces cuproptosis in cancer cells via two synergistic mechanisms. First, CTR1 upregulation enhances copper uptake. In addition, mitochondrial GSH depletion reduces copper chelation capacity. These events collectively elevated mitochondrial copper levels, leading to FDX1/LIAS degradation, DLAT oligomerization, and cell death. Knockout of FDX1 or LIAS conferred radioresistance, while copper chelators or Fe-S cluster stabilization reversed radiotherapy-induced cytotoxicity. Consistently, FDX1-deficient xenografts exhibited diminished radiotherapy efficacy. These findings confirmed that cuproptosis plays a central role in eradicating tumor cells after radiotherapy.

Intriguingly, radioresistant cells and non-responder tumors displayed upregulated metallothioneins (MT1E/X), copper-sequestering proteins that reduce intracellular copper availability and suppress cuproptosis. Mechanistically, radioresistance-associated BTB domain and CNC homolog 1 (BACH1) downregulation alleviated transcriptional repression of MT1E/X, while NRF2 activation further elevated their expression. Restoring BACH1 or depleting NRF2 reversed MT1E/X upregulation and re-sensitized cancer cells to radiotherapy, implicating the coordination of BACH1 and NRF2 as a master regulator of cuproptosis evasion. To counteract MT1E/X-mediated copper sequestration, elesclomol and disulfiram were tested in combination with radiotherapy. These agents synergistically restored mitochondrial copper accumulation, depleted Fe-S/lipoylated proteins, and triggered cuproptosis in radioresistant models. In syngeneic and PDX models, elesclomol combined with radiotherapy achieved near-complete tumor regression, which was dependent on FDX1-mediated cuproptosis.

This study elucidates the mechanism of CTR1/GSH-mediated cuproptosis and the role of MT1E/X in conferring resistance, providing an actionable approach for personalizing radiotherapy. The combination of copper ionophores with radiotherapy represents a clinically viable strategy to overcome radioresistance, especially in cancers with high MT1E/X and low BACH1 expression.

### Regulation of cuproptosis via other mechanisms

In HCC, disulfiram-Cu was shown to trigger dual ferroptosis and cuproptosis, but compensatory upregulation of system x_c_^−^ sustains GSH synthesis, blunting therapeutic efficacy. This resistance arises from cuproptosis-induced endoplasmic reticulum (ER) stress, which stabilizes system x_c_^−^ by inhibiting ubiquitin-proteasome degradation [217]. In castration-resistant prostate cancer (CRPC), enzalutamide enhances mitochondrial dependency, sensitizing cells to copper ionophores via lipoylated protein aggregation and Fe-S cluster destabilization [218]. In bladder cancer (BLCA), YTH N6-methyladenosine RNA binding protein F2 (YTHDF2) destabilizes LIPT1 mRNA via m6A-dependent decay, suppressing lipoic acid metabolism and cuproptosis. Restoring LIPT1 disrupts lipid droplet accumulation and ER homeostasis, thus reactivating cell death [219]. In pancreatic cancer, twist family bHLH transcription factor 1 (TWIST1) stabilizes HK2 by inhibiting its ubiquitination, linking glycolytic flux to cuproptosis [220]. In esophageal squamous cell carcinoma (ESCC), GLS2 depletion synergizes with copper to reprogram TCA cycle and enhance radiosensitivity [221]. In hepatic stellate cells (HSCs), RAS oncogene family member RAB18 suppresses lipophagy and promotes DLD succinylation, thereby triggering cuproptosis. Diallyl trisulfides (DATs) facilitate RAB18 phase separation, selectively inducing HSC death, without damaging normal hepatocytes, which offers a promising strategy for anti-fibrotic therapy [222].

The diverse mechanisms highlighted above illustrate the complex cellular responses to copper stress. They emphasize that the efficacy of cuproptosis induction is not solely determined by copper availability but is profoundly influenced by the cellular context, including metabolic pathways, splicing fidelity, and post-translational modifications. This diversity also reveals multiple ancillary targets that can be exploited to enhance cuproptosis-based therapies across a wide spectrum of cancers.

## Cuproptosis and antitumor immunity

**Fig. 4:**
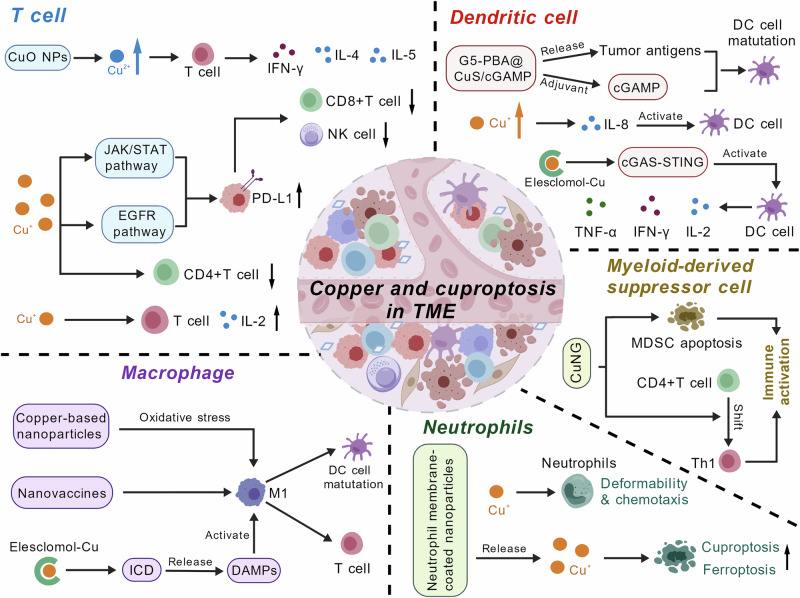
Copper and cuproptosis in the tumor microenvironment. Copper exhibits a biphasic regulatory role in T cells. Moderate copper levels promote T cell activation, exemplified by copper oxide nanoparticles (CuO NPs). However, excessive copper inhibits the activation of both T and NK cells via the JAK/STAT and EGFR signaling pathways. Copper-based nanoparticles, nanovaccines, and elesclomol-Cu promote the polarization of tumor-associated macrophages (TAMs) toward the M1 phenotype, thereby activating T cells and inducing dendritic cell (DC) maturation. Copper boosts neutrophil function by increasing cell deformability and chemotaxis. Neutrophil membrane-coated nanoparticles induce cuproptosis and ferroptosis in tumors while evading immune detection. The copper chelator CuNG induces myeloid-derived suppressor cell (MDSC) apoptosis and shifts CD4^+^ T cells toward Th1 phenotypes, thus enhancing immune activation. Copper-based therapies multifacetedly impact DCs via antigen release, IL-8, and the cGAMP/cGAS-STING pathway to boost antitumor immunity

The intricate interplay between copper metabolism, cuproptosis, and tumor immunity has emerged as a pivotal axis in cancer biology. Copper not only regulates cellular processes but also profoundly influences the tumor microenvironment (TME) and immune responses (Fig. 4). Recent advances highlight the roles of copper homeostasis and cuproptosis in immune cell modulation, immune checkpoint regulation, and therapeutic strategies leveraging copper’s immunogenic potential [223, 224]. CTR1 overexpression was found to correlate with poor survival in breast, cervical, head and neck, and esophageal cancers [225], accompanied by increased infiltration of immunosuppressive cell types (e.g., Tregs, M2 macrophages) and upregulation of PD-L1/CTLA4 [226, 227]. Conversely, FDX1 seemingly plays a contrasting role, as its high expression in colorectal cancer (CRC) predicts better survival and CD8^+^ T cell infiltration, while downregulation in clear cell renal cell carcinoma (ccRCC) correlates with impaired immunity and worse prognosis [228, 229]. These findings suggest cuproptosis-related genes as potential biomarkers for immune stratification.

### Copper and immune cell dynamics in the tumor microenvironment

Copper plays a dual role in T cell biology. While appropriate copper levels are essential for T cell proliferation and interleukin-2 (IL-2) production [230, 231], excess copper can promote immunosuppression [232]. Copper chelators, such as TTM, reduce intratumoral copper levels, thereby downregulating PD-L1 expression via the inhibition of the JAK/STAT and EGFR pathways. This results in increased CD8^+^ T cell and natural killer (NK) cell infiltration, consequently improving survival in murine models [232]. Additionally, diminished copper levels are associated with reduced tumor vessel diameter and endothelial cell proliferation and activation, and increased CD4^+^ T cell infiltration [233]. Conversely, copper oxide (CuO) nanoparticles stimulate T cell proliferation and T helper type 1 (Th1)/Th2 cytokine secretion, including IFN-γ, IL-4, and IL-5, suggesting context-dependent immunomodulatory effects [234]. Regulatory T cells (Tregs), key mediators of immune tolerance, are suppressed in tumors with upregulated cuproptosis-related genes like LIPT1, which correlates negatively with Treg infiltration and positively with PD-L1 expression [235]. These findings emphasize the complex role of copper in reshaping T cell landscapes to modulate tumor immunity.

Tumor-associated macrophages (TAMs) are central to TME immunosuppression. Copper modulates macrophage polarization, favoring pro-inflammatory M1 phenotypes under certain conditions. For instance, copper-based nanoparticles (which is discussed in a separate section) such as CuTCPP@MOF nanodots@Mannosamine induce cuproptosis and ferroptosis in macrophages via oxidative stress, thus eliminating intracellular pathogens and enhancing antimicrobial responses [236]. Copper ionophores like elesclomol trigger immunogenic cell death (ICD) to release damage-associated molecular patterns (DAMPs) that recruit and activate macrophages, thereby promoting antigen presentation and T cell priming [237]. Nanovaccines such as Cu-THBQ/AX exploit copper’s ability to convert M2-like TAMs to antitumor M1 phenotypes, enhancing dendritic cell (DC) maturation and cytotoxic T lymphocyte (CTL) infiltration [238]. These strategies underscore copper’s potential to reverse TAM-mediated immunosuppression.

Copper deficiency has been found to impair neutrophil function, thus reducing deformability and chemotaxis [239]. Interestingly, engineered nanoparticles like MetaCell utilize neutrophils to deliver copper-loaded liposomes, inducing cuproptosis and ferroptosis in tumors while evading immune detection [240]. Myeloid-derived suppressor cells (MDSCs), critical suppressors of antitumor immunity, are targeted by copper chelators such as CuNG, which induce MDSC apoptosis and shift CD4^+^ T cells toward Th1 phenotypes, leading to immune activation [241]. These findings position copper as a key regulator of myeloid cell plasticity in the TME.

Copper’s impact on DCs is multifaceted. It has been suggested that copper may affect DC activation and inflammatory responses by increasing IL-8 production [242]. Elesclomol-Cu can enhance DC activation by stimulating the cGAS-STING pathway, leading to increased secretion of IL-2, TNF-α, and IFN-γ, which promotes CTL responses [243]. The copper-based nanovaccine G5-PBA@CuS/cGAMP leverages photothermal effects to release tumor antigens and adjuvant cGAMP, driving DC maturation and systemic antitumor immunity [244]. Such approaches highlight copper’s ability to amplify DC-mediated antigen presentation, which is a key step of adaptive immunity.

### Copper induction of immunogenic cell death

Immunogenic cell death (ICD) is a form of regulated cell death that activates the immune system by releasing danger signals, such as DAMPs, from dying cells. These signals recruit and activate DCs, which prime T cells to recognize and eliminate tumor cells, thereby enhancing antitumor immunity [245]. Unlike non-immunogenic apoptosis, ICD transforms cell death into a systemic immune response. This is critical for therapies, such as chemotherapy, radiotherapy, and targeted therapy, to achieve durable cancer control. Cuproptosis has emerged as a crucial mechanism to induce ICD for eradicating cancer cells. Central to this process is the ability of copper ions to disrupt mitochondrial function, trigger oxidative stress, and release DAMPs, which ultimately activates systemic antitumor immune responses. Recent studies across diverse cancer types have illuminated the dual role of cuproptosis – direct tumor cell elimination and immune system activation – providing new avenues to combat malignancies.

In CRC, copper overload induces ER stress to prompt the release of DAMPs, such as ATP and high-mobility group box 1 (HMGB1). These signals orchestrate DC maturation and recruit CTLs and M1 macrophages into the TME, transforming immunologically “cold” tumors into inflamed niches. To amplify this effect, an in situ cuproptosis-inducing system was developed, which synergizes with the agonist of toll like receptor 7 (TLR7), Imiquimod, to further enhance immune cell infiltration and tumor suppression. This approach not only validates cuproptosis as a potent ICD inducer but also underscores its potential to overcome the immunosuppressive barriers prevalent in CRC [246].

The therapeutic potentials of traditional copper ionophores have inspired the development of novel agents. YL21, a naphthoquinone-based compound with dual dithiocarbamate groups, exemplifies this innovation. Unlike disulfiram, YL21 exhibits superior solubility and efficacy when complexed with copper ions. It disrupts mitochondrial respiration, promotes lipoylated DLAT oligomerization, and induces oxidative and ER stress, consequently leading to ICD. In breast cancer models, YL21-Cu complexes significantly inhibit tumor growth while activating antitumor immunity, making it a promising candidate for clinical translation [247].

Advancements in nanotechnology have enabled precision copper delivery. Nanoplatforms encapsulating elesclomol and Cu^2+^ release copper ions in response to intracellular conditions, generating mitochondrial ROS and triggering cuproptosis. In fibrosarcoma, these nanoparticles induce ICD, activate DCs, and enhance CD8^+^ T cell infiltration, thus achieving robust tumor regression. Its tumor-specific release mechanism minimizes off-target effects while maximizing immunogenic outcomes, highlighting their potential for personalized therapy [248].

Mitochondria-directed strategies have been developed to achieve more precise copper targeting. For example, the triphenylphosphonium-modified copper complex TPP-CuET accumulates in mitochondria and disrupts the TCA cycle, ATP synthesis, and electron transport, thus amplifying immunogenic cuproptosis. This mitochondrial targeting enhances copper uptake by 50% compared to non-targeted analogs, leading to pronounced DAMP release and M1 macrophage polarization. Transcriptomic analyses reveal that TPP-CuET upregulates MHC-I-mediated antigen presentation to activate CTLs and NK cells, thereby bridging metabolic disruption and immune activation [237].

Collectively, these studies underscore cuproptosis as an inducer of ICD in cancer. By harnessing copper’s redox properties, advanced ionophores, and smart nanocarriers, researchers are unlocking dual therapeutic benefits, direct tumor cell killing and TME reprogramming. This evolving paradigm not only redefines copper’s role in different forms of RCD but also heralds a new era of metal-ion-based cancer immunotherapy.

### Role of cuproptosis in cancer immunotherapy

Immune checkpoint blockade (ICB) is a cancer immunotherapy strategy that disrupts inhibitory signaling pathways (e.g., PD-1/PD-L1) to reactivate T-cell-mediated antitumor immunity, effectively overcoming tumor immune evasion and demonstrating clinical success across multiple cancer types. Copper metabolism directly influences PD-L1 expression. In NSCLC, disulfiram-Cu complexes upregulate PD-L1 via HIF-1 signaling, fostering immune evasion [249]. Conversely, copper chelation reduces PD-L1 levels by promoting its ubiquitination and degradation, thereby enhancing T cell infiltration [232]. A biomimetic nanoplatform (CuX-P) has demonstrated potentials for the treatment of TNBC by integrating cuproptosis induction, photothermal therapy, and immunotherapy. CuX-P specifically targets PD-L1 on tumor cells, facilitating dual internalization of both the nanoplatform and PD-L1 via a “patch-like” recognition mechanism, resulting in the depletion of PD-L1 on cell surface and the enrichment of copper in tumor cells [250]. Interestingly, however, another study showed that the copper chelator JYFY-001 enhance PD-1 blockade efficacy in CRC [251]. Therefore, the effect of copper on T cell activation could be context-dependent.

Besides PD-1/PD-L1, copper modulates other checkpoints. CD47, a “don’t eat me” signal, is targeted by biomimetic nanoparticles, such as CD47@CCM-Lap-CuS, that evade macrophage phagocytosis while delivering copper for photothermal therapy [252]. In HCC, disulfiram-Cu synergizes with CD47 blockade to enhance DC maturation and CD8^+^ T cell cytotoxicity [253]. Moreover, copper nanocomplexes combined with anti-CTLA-4 antibodies amplify antitumor effects in melanoma models, revealing copper’s versatility in checkpoint modulation [254].

In addition to ICB, CAR-T therapy is a cutting-edge cancer immunotherapy that genetically engineers a patient’s T cells to express chimeric antigen receptors (CARs), leading to targeted recognition and destruction of cancer cells. This approach has demonstrated remarkable efficacy in the treatment of hematologic malignancies, such as leukemia and lymphoma. Copper ionophores combined with radiotherapy have been found to reprogram CAR-T cells, thus overcoming stromal barriers in breast cancer models. This study presents a groundbreaking non-genetic strategy that enhances CAR-T efficacy against solid tumors through copper overload and irradiation, boosting CAR-T functionality and reversing immunosuppressive TME [255].

Despite the potential of cuproptosis in immunomodulation, significant challenges remain due to the complex TME. Hypoxia within the TME suppresses OXPHOS, thereby inhibiting cuproptosis efficacy. To counteract this, innovative approaches such as oxygen-generating nanoparticles (e.g., CAT-ecSNA-Cu) have been developed to sensitize tumors by modulating metabolic conditions [256]. Additionally, the lack of immune cell specificity carries risks, as copper therapies may affect normal immune cells, possibly resulting in unexpected adverse events. Biomarker development also lags behind clinical needs. While cuproptosis-related genes, such as FDX1 and LIPT1, demonstrate prognostic potential, their clinical utility requires rigorous validation in diverse patient cohorts to enable precise patient stratification [235, 257]. Hence, advancing this field will demand a deeper understanding of cuproptosis-immune system interactions and refinement of nanocarrier designs for optimized delivery.

## Targeting cuproptosis for cancer therapy

Cuproptosis offers a novel strategy for cancer treatment. Central to this approach are copper ionophores, such as elesclomol and disulfiram, that selectively shuttle extracellular copper into cancer cells. In addition, nanoparticle-based systems are advancing precision delivery. These nanocarriers improve tumor targeting, reduce systemic toxicity, and allow for controlled copper release in the tumor microenvironment.

### Copper ionophores

Disulfiram, a U.S. Food and Drug Administration (FDA)-approved aldehyde dehydrogenase (ALDH) inhibitor traditionally used for alcohol dependence, has garnered significant attention in recent years for its potential as a copper ionophore in anticancer therapy [258–261]. Mechanistically, disulfiram binds to copper to form the metabolite bis-diethyldithiocarbamate-copper (CuET), which facilitates copper transport into cancer cells, triggering multiple cell death pathways, including apoptosis, ferroptosis, and cuproptosis [39, 262, 263]. Its antitumor activity also involves targeting the NPL4 subunit of the p97 segregase to disrupt the ubiquitin-proteasome system [258], thus activating stress-related signaling pathways, such as JNK/p38 and NF-κB [264–267], and inducing oxidative stress via ROS generation [268–270].

Preclinical studies reveal disulfiram-Cu’s synergistic effects with conventional therapies [40, 41]. disulfiram-Cu complexes have demonstrated efficacy in overcoming resistance to cisplatin [271], paclitaxel [259], gemcitabine [266], and other chemotherapeutic agents [267, 272, 273]. Furthermore, disulfiram-Cu selectively eliminates ALDH^+^ CSCs, a population implicated in the resistance to chemotherapy and radiotherapy [259, 274]. Combined with ionizing radiation, disulfiram-Cu induces ICD characterized by HMGB1 release, promoting CAR-T expansion and suppressing metastatic breast cancer growth in murine models [255]. However, clinical outcomes remain inconsistent. A phase II trial in advanced NSCLC reported improved median overall survival (OS) with disulfiram-Cu plus cisplatin/vinorelbine (10.0 vs. 7.1 months), including exceptional long-term survival in two stage IV patients [261]. Conversely, a phase II trial in relapsed germ cell tumors showed no restored cisplatin sensitivity, with median progression-free survival (PFS) and OS of only 1.4 and 2.9 months, respectively [275]. Similarly, a phase II-III trial in recurrent glioblastoma revealed no significant 6-month OS benefit from disulfiram-Cu combined with alkylating chemotherapy [276].

Ongoing clinical trials aim to optimize disulfiram’s therapeutic potential, such as combining it with gemcitabine in metastatic pancreatic cancer (NCT02671890) or integrating disulfiram-Cu into perioperative regimens for newly diagnosed glioblastoma (NCT02715609). Key challenges include precise modulation of intratumoral copper homeostasis, as maintaining elevated copper levels in patients remains technically challenging. Additionally, the nonspecificity of copper ionophores, such as disulfiram’s concurrent effects on iron metabolism, may contribute to off-target toxicity. Future directions should focus on developing tumor-specific copper ionophores and identifying predictive biomarkers to stratify patient populations likely to benefit from cuproptosis-based therapies. Addressing these limitations could reposition disulfiram as a versatile agent in overcoming therapeutic resistance and improving outcomes in aggressive malignancies.

Elesclomol (STA-4783), a highly lipophilic copper ionophore, binds to extracellular Cu^2+^ to form a complex that facilitates copper transport into tumor cells, triggering multiple modes of cell death, including cuproptosis [39]. Its mechanisms involve multi-pathway regulation in addition to the canonical cuproptosis. The elesclomol-Cu complex elevates ROS levels, which triggers oxidative stress and promotes copper-dependent degradation of ATP7A, thereby disrupting intracellular copper homeostasis [277, 278]. Recent studies suggest that its cuproptotic effects may extend beyond reliance on the mitochondrial protein FDX1. It could potentially deliver copper to non-mitochondrial cuproproteins to activate non-canonical death pathways, though precise mechanisms require further validation [279].

Preclinical evidence reveals significant anticancer activity of elesclomol-Cu in cisplatin-resistant lung cancer cells, vemurafenib-resistant melanoma cells, and proteasome inhibitor-resistant breast cancer cells, which is mediated by elevated mitochondrial metabolism in these resistant populations [280]. Intriguingly, chemotherapeutic agents such as APG115 and PX-478 may enhance tumor sensitivity to elesclomol, providing combinatorial therapeutic strategies [88, 171]. Unfortunately, elesclomol has not yet shown effective therapeutic outcomes in clinical trials. A randomized phase II trial in pretreated melanoma patients showed that elesclomol combined with paclitaxel significantly prolonged median PFS (112 vs. 56 days), reducing the risk of disease progression or death by 41.7%, with a trend toward improved median OS (11.9 vs. 7.8 months) [281]. However, a subsequent phase III trial in advanced melanoma failed to meet its primary PFS endpoint. A predefined subgroup analysis revealed PFS benefits in a specific patient group: those with baseline serum lactate dehydrogenase (LDH) levels within the normal physiological range [282]. This also highlights the potential influence of tumor metabolic status (e.g., glycolysis activity) on cuproptosis sensitivity – low LDH levels correlate with impaired glycolysis and enhanced mitochondrial metabolism, thereby fostering the metabolic dependencies of cuproptosis. Also, a phase II study in platinum-resistant ovarian cancer reported an objective response rate of 19.6%, median PFS of 3.6 months, and OS of 13.3 months with elesclomol/paclitaxel combination, which is comparable to paclitaxel monotherapy [283].

Given the limited success of existing elesclomol-based therapies in clinical studies, there is a clear need for exploring novel combination approaches in future trials. In addition, the multi-modal cell death mechanisms of elesclomol require clarification of crosstalk between cuproptosis and other RCD pathways (e.g., apoptosis, ferroptosis) to optimize therapeutic targeting. Future direction should also prioritize patient stratification using biomarkers such as the levels of p53, MT2A, or LDH, the hypoxic or redox status, and mutations in *ARID1A* or *SF3B1*, in which glycolytic remodeling or copper homeostasis serves as determinants of therapeutic efficacy.

In addition to disulfiram and elesclomol, bis(thiosemicarbazones), as a group of copper ionophores, have attracted significant attention due to their unique copper-binding properties and anticancer activity [284]. Among them, diacetyl-bis(N4-methylthiosemicarbazone) (ATSM) and glyoxal-bis(N4-methylthiosemicarbazone) (GTSM) exhibit distinct mechanisms of action despite their structural similarities. Structure-activity relationship (SAR) studies revealed that GTSM, with its unsubstituted diimine backbone, maintains high antiproliferative activity, whereas the methylated diamine backbone of ATSM decreases the reduction capacity of its copper complex (ATSM-Cu) [285]. In vitro experiments demonstrated that GTSM-Cu shows superior cytotoxicity against prostate cancer PC3 cells compared to ATSM-Cu. Moreover, supplementing the culture medium with physiological concentrations of copper (20 μM) further reduces the LD50 of Cu-GTSM from 1.5 μM to 150 nM [286]. Mechanistically, GTSM releases copper ions in the reductive tumor microenvironment, selectively killing cancer cells with high ROS and low GSH levels [287]. In contrast, ATSM exhibits weaker anticancer effects due to its limited copper release. This selectivity may be attributed to the high copper accumulation and redox imbalance in tumor tissues.

Among the hydroxyquinoline (HQ) class of copper ionophores, 7-iodo-5-chloro-8-hydroxyquinoline (CQ) is a representative compound [284]. By forming a complex with copper (CQ-Cu), it targets the proteasome [288, 289] and induces the degradation of X-linked inhibitor of apoptosis protein (XIAP) [290], thereby relieving caspase inhibition and triggering cancer cell apoptosis. Notably, Cu-CQ selectively triggers XIAP depletion in prostate cancer cells, without affecting normal prostate epithelial cells, indicating its tumor-specific toxicity. However, the clinical application of CQ is limited by its broad-spectrum toxicity (e.g., neuropathy) [291]. To optimize efficacy and safety, research has shifted toward developing derivatives such as PBT2 [292] and nitroxoline [293]. These derivatives exhibit enhanced antiproliferative activity in the presence of copper, potentially by altering intracellular copper distribution or modifying mechanisms of action to improve therapeutic outcomes and reduce cytotoxicity.

In summary, copper ionophores demonstrate promising anticancer potential by disrupting copper homeostasis and inducing multiple cell death pathways, yet clinical efficacy remains to be improved. Future research should focus on optimizing combination therapies, refining tumor-specific delivery, and identifying predictive biomarkers to enhance therapeutic precision. Addressing these challenges could unlock the full potential of copper ionophores in overcoming treatment resistance and improving outcomes in aggressive cancers.

### Nanomedicine

The emergence of cuproptosis has revolutionized therapeutic strategies for cancer treatment. Nanotechnology is key to harnessing its potential. Various approaches have been developed for the intracellular delivery of copper ions, including inorganic nanoparticles, metal-organic frameworks (MOFs), biomimetic delivery systems, hydrogels, and polymer-based carriers [294].

Copper inorganic nanoparticles have gained significant attention due to their tunable physicochemical properties and multifaceted biological effects. These nanomaterials, classified by oxidation states (e.g., Cu^2+^ and Cu^+^), exhibit distinct mechanisms in triggering cuproptosis. Cu^2+^-based compounds like CuO nanoparticles leverage lysosomal acidity to release copper ions and payloads [295], while CuS nanofibers demonstrate exceptional photothermal conversion attributed to their cauliflower-like surface morphology [296]. Further advancements include Cu-BiSex nanocrystals with increasing photothermal efficiency and peroxidase-like activity for synergistic ROS generation, apoptosis and cuproptosis [297]. However, therapeutic translation faces challenges, particularly low tumor delivery efficiency (<1%) due to rapid clearance, nonspecific distribution, tumor heterogeneity, lack of surface modifications and targeting ligands, and biological barriers [298, 299]. Innovative solutions such as near-infrared light-controlled nanomotors [300] and tumor microenvironment-responsive Cu_2_(PO_4_)(OH) nanoparticles, which are degraded into ultrasmall Cu_9_S_8_ in H₂S-rich environments [301], show promise in enhancing cellular uptake and specificity.

Cu^+^ compounds like Cu_2_O nanoparticles exploit elevated CTR1 expression and ROS-mediated ATPase inhibition to increase intracellular copper and effectively promote cuproptosis [302]. Microfluidically synthesized CuH nanoparticles provided improved purity and solubility, while copper nanocatalysts with peroxidase/catalase activities remarkably suppress tumors via catalytic therapy and cuproptosis [303, 304]. A radiation-responsive copper-containing nanocapsule-like polyoxometalate was developed to release copper ions upon irradiation [305]. This strategy triggered cuproptosis, overcoming acquired radioresistance at clinical radiation doses and inducing abscopal effects. The approach synergistically enhances radiotherapy’s local efficacy and systemic antitumor immunity, offering a novel solution for tackling radioresistance. Despite these advances, challenges persist in controlling nanoparticle distribution and metabolism. Additionally, the potential cytotoxicity at high doses requires careful optimization of dosing regimens. The field is now shifting toward sophisticated nano-delivery systems to address these limitations, focusing on spatial-temporal control and targeted release mechanisms [294].

Metal-organic frameworks (MOFs) represent a breakthrough in copper delivery, which integrates high porosity with stimuli-responsive degradation [306]. MOF-199, containing higher level of copper, can be degraded in acidic tumor microenvironments to release Cu^2+^ that is subsequently reduced to more toxic Cu^+^ by FDX1 [307, 308]. It can be modified with hyaluronic acid or chondroitin sulfate, which allows it to bind to tumor surface receptors, thereby enhancing the delivery efficiency and tumor targeting capability [309, 310]. 2D MOFs exhibit biomedical potential due to their ultrathin structure, large surface area, and pH-/ultrasound-responsive properties. For example, these nanosheets show anticancer effects by generating ROS via FDX1-mediated Cu^2+^ reduction to Cu^+^, enhancing chemotherapy efficacy [311, 312]. Interestingly, they have been shown to display high tumor specificity with limited toxicity to normal cells (e.g., 293 T cells). In addition, copper-doped zeolitic imidazole framework-90 (ZIF-90) and chiral D-/L-Cu_x_OS@Fe-MOF nanoparticles exhibit the potential for both copper delivery and GSH depletion, thereby inducing cuproptosis and ferroptosis [313, 314].

Biomimetic delivery systems have emerged as powerful tools to evade immune clearance. Cancer cell membrane-coated nanoparticles exploit homotypic targeting; therefore, 4T1 membrane-cloaked PCD@CM shows enhanced tumor accumulation [315]. Immune cell mimics, including PD-L1-overexpressing T-cell membrane coatings, create positive feedback loops for nanoparticle internalization [250], while neutrophil membrane modifications extend the half-life of blood circulation [316]. Bacterial membrane disguises, such as E. coli-coated nanoparticles, leverage tumor-associated antigens for colorectal cancer targeting [317]. Collectively, biomimetic delivery systems improve therapeutic precision and immune evasion by integrating natural membrane properties to enhance targeting, prolong circulation, and modulate immune interactions.

Smart hydrogel systems address the need for sustained, environmentally responsive copper release. pH-sensitive elesclomol-Cu-alginate hydrogels demonstrate five-fold slower drug release at acidic tumor microenvironment, reducing systemic toxicity [318]. More sophisticated multi-responsive hydrogels, like the Cu^2+^/NCTD/GA ternary system, exhibit shear-thinning and self-healing properties with laser-triggered drug release capabilities [319]. These injectable formulations adapt to dynamic tumor microenvironments, though challenges remain in large-scale production and long-term safety assessment.

Polymer-based carriers provide additional versatility in copper delivery. PAMAM dendrimers can remarkably bolster copper loading through surface amino groups, resulting in an increase in intracellular copper accumulation and cuproptosis [320, 321]. In addition, PEGylation strategies, which involve the application of polyethylene glycol (PEG) to coat nanoparticles, prolong circulation time while also limiting cellular uptake [322, 323]. Recently, a self-amplifying ROS-responsive nanoplatform (ECPCP) is developed with a polymer based on cinnamaldehyde and polyethylene glycol that encapsulates elesclomol-Cu compound. These nanoparticles mitigate spatial barriers and improve cellular uptake, thereby inducing cuproptosis and ICD [324]. Furthermore, emerging amphiphilic polymers, a macromolecule that possesses both hydrophilic and lipophilic properties, with their ROS-sensitive designs, represent promising alternatives for elesclomol-Cu encapsulation and targeted delivery [325, 326].

The convergence of these nanotechnological approaches has significantly advanced cuproptosis-based therapies, yet critical challenges remain. For further reading, readers are directed to relevant reviews on related topics [294, 327, 328]. Precise control of nanoparticle pharmacokinetics, mitigation of off-target cytotoxicity, and comprehensive metabolic studies are essential for clinical translation. Future directions should focus on multi-omics-guided nanoparticle design, combinatorial regimens with immunotherapy, and biomarker development for patient stratification. As the understanding of copper homeostasis in cancer grows, these nano-delivery platforms possess immense potential to transform cuproptosis from a mechanistic novelty into a clinically viable therapeutic paradigm.

## Concluding remarks

The discovery of cuproptosis has unveiled a paradigm-shifting mechanism of RCD, driven by mitochondrial copper overload and metabolic vulnerability. Cuproptosis is driven by the aggregation of lipoylated TCA cycle enzymes and destabilization of Fe-S cluster proteins, thereby establishing mitochondrial respiration and copper homeostasis as central determinants of cellular fate. Central to cuproptosis is the intricate interplay between copper homeostasis, metabolic reprogramming, and cellular signaling networks. Key regulators such as p53, HIF-1α, Wnt/β-catenin, and AKT dynamically modulate cuproptosis sensitivity by rewiring glycolytic flux, mitochondrial respiration, and copper homeostasis. These findings not only expand our understanding of copper’s dual roles as an essential micronutrient and cytotoxic agent but also provide a unique therapeutic avenue for malignancies characterized by metabolic dysregulation.

Translational efforts have focused on copper ionophores and nanotechnology-driven delivery systems to exploit cuproptosis. While ionophores demonstrate preclinical efficacy, clinical outcomes remain inconsistent, highlighting challenges such as narrow therapeutic windows, metabolic heterogeneity, and compensatory resistance mechanisms. Nanomedicine innovations aim to enhance tumor-specific copper delivery, yet their long-term safety and clinical translatability require further optimization. Combinatorial strategies, such as pairing copper ionophores with p53 agonists, HIF-1α inhibitors or radiotherapy, show promise in overcoming resistance by synchronizing metabolic vulnerability with copper toxicity.

Although studies reveal the regulation of cuproptosis by individual pathways, a critical and largely unexplored dimension is the crosstalk between these regulators within the complex tumor microenvironment. For instance, how does a hypoxic tumor with wtp53 integrate conflicting signals? While HIF-1α activation promotes glycolytic flux and copper chelation to suppress cuproptosis, p53 may simultaneously attempt to sustain mitochondrial metabolism, which induces cuproptosis sensitivity. The eventual outcome likely depends on the relative strength and context of these signals. Similarly, the AKT-mediated phosphorylation and inactivation of FDX1 may directly oppose p53’s efforts to enhance mitochondrial function. Elucidating these dynamic interactions will be essential for predicting patient responses and designing effective combination therapies.

Key questions remain for future study. First, the crosstalk between cuproptosis and other RCD pathways (e.g., apoptosis and ferroptosis) warrants further exploration. This may lead to the development of synergistic regimens that prevent compensatory survival mechanisms. Second, biomarker development is essential for patient stratification. Future efforts should focus on integrated panels that capture the complexity of the cuproptosis regulatory network. These panels could assess the tumor’s metabolic state (e.g., glycolytic vs. OXPHOS), copper homeostasis (e.g., ATP7B/CTR1 expression), genetic landscape (e.g., *ARID1A*, *SF3B1*, *TP53* status), and TME context (e.g., HIF-1α activity, immune cell infiltration). Third, the role of copper in tumor immunity, particularly its impact on T-cell function and checkpoint regulation, remains underexplored. Finally, advancing precision delivery systems, which minimizes off-target effects and maximizes intratumoral copper accumulation, is critical to realizing clinical translation. By addressing these challenges, cuproptosis-based therapies could be a pivotal component of precision oncology, providing novel solutions for cancers refractory to conventional treatments.
